# Nutritional Adequacy of Flour Product Enrichment with Iodine-Fortified Plant-Based Products

**DOI:** 10.3390/nu16244261

**Published:** 2024-12-10

**Authors:** Agata Jankowska, Krystyna Szymandera-Buszka

**Affiliations:** Department of Gastronomy Science and Functional Foods, Faculty of Food Science and Nutrition, Poznań University of Life Sciences, Wojska Polskiego 31, 60-624 Poznań, Poland

**Keywords:** iodine sources, stability of iodine, food fortification, flour products, iodine deficiencies

## Abstract

This study assessed the nutritional value of designed vegan flour products (Gnocchi and Ciabatta) by adding iodine-fortified dried vegetables. The KI and KIO_3_ constituted the sources of iodine. The pumpkin, cauliflower, carrot, broccoli and beetroot were used as a matrix for the iodine applied. The nutritional value was tested based on iodine content and antioxidant activity. The content of thiamine was determined in samples of Ciabatta rolls. The antioxidant activity of Gnocchi dumplings and Ciabatta rolls was analysed before and after heat treatment (baking and cooking) and after storage. It was confirmed that the designed cereal products (Ciabatta rolls and Gnocchi dumplings) with the addition of iodine-fortified dried vegetables are a good source of iodine in the diet and can be used as an element of IDD prevention. Consuming 100 g of Ciabatta rolls (1 pc.) provides coverage of iodine demand at the level of ~6% RDA, while 100 g of Gnocchi dumplings (20 pcs.) provides ~19% RDA. The type of iodine carrier (KI or KIO_3_) and type of vegetable for iodine introduced into cereal products affect the content of selected nutritional value indicators, such as iodine and thiamine content and antioxidant properties of the product.

## 1. Introduction

It is broadly understood that flour products have been an essential element of people’s diets in many parts of the world for centuries. Products containing flour, especially wheat, corn and rice flour, are the basis of the culinary traditions of many cultures [1]. Additionally, they are often cheap, filling and easy to prepare, contributing to their great popularity among societies at various stages of economic development. Although flour products are considered traditional, the increased consumer interest in food with high nutritional value, which provides additional health benefits, forces food producers to constantly look for new opportunities to improve their recipes [2]. Ways to increase the health-promoting effects of flour products include reducing the share of salt and increasing the share of dietary fibre in these products [3]. Therefore, enriching flour products with vegetables could be a nutritional alternative to improve health.

Additionally, vegetables are a source of nutrients and bioactive ingredients, such as vitamins, minerals and phenolic compounds [4,5,6,7,8,9,10]. According to the latest dietary guidelines, they should constitute the basis of a healthy diet. The World Health Organization recommends that adults consume at least two portions of fruit and three portions of vegetables per day [11,12,13]. The enrichment of flour products with vegetables will allow for an increase in the effectiveness of the overall profile of the diet [4,13,14,15,16,17,18,19]. Despite the health benefits, vegetable consumption remains too low. According to Wallace et al. 2019 [13], only 10% of Americans meet the recommendations for vegetable consumption [4]. Preliminary studies also confirm using selected vegetables as a nutrient matrix. This also applies to iodine [20]. Research has confirmed the possibility of using pumpkin, carrot, broccoli, cauliflower and beetroot as the matrix for iodine. It has been shown that iodine reproducibility was at level 68–95% after drying.

Preliminary data confirm the possibility of enrichment of a ready-to-eat food product, e.g., flour products [21].

Enriching flour products with iodine-fortified vegetables can diversify the diet and also be an effective alternative source of iodine for all consumers. This may especially apply to people who are overweight and vegetarians, especially vegans.

Iodine is a difficult element to provide in adequate amounts in the daily diet due to its varied environmental content and, consequently, in food products [22]. At the same time, it is crucial for the functioning of the thyroid gland, and indirectly for proper psychosomatic development, functioning of metabolism and proper cognitive functions [23]. Therefore, the deficiency of this nutrient is a serious public health problem in more than 120 countries around the world [24]. One of the most popular and considered the most effective is the strategy of fortifying table salt with iodine by adding potassium iodide or iodate. This method is recommended by WHO [25,26]. Although this method is cheap, quick and simple to carry out [27], it may pose some technological and nutritional difficulties.

Potassium iodate (KIO_3_) is quite stable and has a longer shelf life than potassium iodide (KI). Potassium iodide (KI) is known as a hygroscopic substance that has a short shelf life at elevated temperatures and humidity due to the hydrolytic loss of iodine vapours [28]. The stability of these compounds should be taken into account when planning food fortification with their participation. Due to its higher stability, iodate is most often indicated as a more adequate substance for fortification [29,30]. However, in addition to the stability of these substances, their possible effects on other food components should also be considered. Iodide is a strong reducing agent, and iodate is a strong oxidising agent [29,31]. Possible reactions that iodine and its salts could cause in food include increased oxidative reactions and reduced bioavailability of other nutritionally important substances [31]. Iodate’s ability to produce reactive oxygen species due to its oxidising properties [32] is undesirable, especially in the current trend of designing functional foods with high antioxidant potential.

Due to its oxidation, iodine introduced into products increases thiamine losses in both a free and bound form. The type of iodine salt carrier is important because thiamine losses were significantly greater when introducing iodine with iodised table salt than with an iodised collagen preparation [33,34,35]. This study aimed to assess the nutritional value of designed vegan flour products with the addition of iodine-fortified dried vegetables. Due to its oxidation, iodine introduced into the product may also increase thiamine losses. The type of iodine salt carrier is important because thiamine losses were significantly greater when introducing iodine with iodised table salt than with an iodised collagen preparation [33,34].

Iodine-enriched food products should improve rather than lower the health-promoting properties of fortified food [36]. Therefore, this study aimed to assess the nutritional value of designed vegan flour products (Gnocchi and Ciabatta) with the addition of iodine-fortified dried vegetables.

It was hypothesised that the different vegetable varieties affect iodine stability in a ready-to-eat flour product of the enriched dried product and its antioxidant activity.

## 2. Materials and Methods

### 2.1. Material

KI and KIO_3_ constituted the sources of iodine (Merc, Darmstadt, Germany). The Muscat pumpkin (*Cucurbita maxima* Duch.), cauliflower (*Brassica oleracea* var. *botrytis* L.), carrot (*Daucus carota* L.), broccoli (*Brassica oleracea* L.) and beetroot (*Beta vulgaris* L.) were used as a matrix for the iodine applied. The products were purchased in the retail trade (Poznań, Poland).

#### 2.1.1. Carriers Preparation

The preparation of vegetables for fortification proceeded according to the procedure presented in the publication by Zaremba et al. 2022 [20]. Detailed impregnation conditions were described in the publication Jankowska et al.’s 2023 publication [37].

#### 2.1.2. Products Formulations

##### Gnocchi Dumplings Formulations

Gnocchi dumplings were made using the analysed carriers. Potatoes and flour were purchased in the retail trade. The potatoes were washed under running tap water and cooked in a stainless-steel pot using boiling tap water (1:2 (*m*/*v*)) for 25 min at 100 °C until soft. Then, the potatoes were drained and cooled at room temperature (20 ± 2 °C) for 15 min. The peeled potatoes were mashed using a ricer (Westmark GmbH, Lennestadt-Elspe, Germany). The following dumpling variants were prepared:

- Potato 80%; wheat flour (10%); water (10%) (GWN) and KIO_3_ (GWN_KIO_3_) or KI (GWN_KI);
- Potato 56%; wheat flour (10%); water (10%) and pumpkin non-iodised (GP_NI) or KIO_3_ (GP_KIO_3_) or KI (GP_KI)
- Potato 56%; wheat flour (10%); water (10%) and carrot non-iodised (GCR_NI) or KIO_3_ (GCR_KIO_3_) or KI (GCR_KI)
- Potato 56%; wheat flour (10%); water (10%) and cauliflower non-iodised (GCF_NI) or KIO_3_ (GCF_KIO_3_) or KI (GCF_KI)
- Potato 56%; wheat flour (10%); water (10%) and broccoli non-iodised (GB_NI) or KIO_3_ (GB_KIO_3_) or KI (GB_KI)
- Potato 56%; wheat flour (10%); water (10%) and beetroot non-iodised (GBT_NI) or KIO_3_ (GBT_KIO_3_) or KI (GBT_KI)

Preparations impregnated with iodine were added to the batter in the hydrated form (at 1:3 ratios). After a thorough mixing of the additives (approximately 10 min), pieces with a typical oval shape and similar weights (approximately 5 g) were formed [21].

##### Ciabatta Roll Formulations

As with the Gnocchi, all the products needed to prepare the dough for the Ciabatta rolls were purchased in the retail trade.

To prepare the dough, wheat flour, water, instant yeast, non-iodised salt and iodine-enriched dried vegetables were used. The following Ciabatta roll variants were prepared:

- Wheat flour (57%); water (41.8%); instant yeast (0.9%) and salt (0.3%)—non-iodised salt (BWN), %); KIO_3_—iodised salt (BWN_KIO_3_) or KI-iodised salt (BWN_KI);
- Wheat flour (52%); water (41.8%); instant yeast (0.9%); and pumpkin-impregnated with salt (5%) non-iodised (BPN), KIO_3_ (BPN_KIO_3_) or KI (BPN_KI);
- Wheat flour (52%); water (41.8%); instant yeast (0.9%); and carrot-impregnated with salt (5%) non-iodised (BCRN), KIO_3_ (BCRN_KIO_3_) or KI (BCRN_KI);
- Wheat flour (52%); water (41.8%); instant yeast (0.9%); and cauliflower-impregnated with salt (5%)—non-iodised (BCFN), KIO_3_ (BCFN_KIO_3_) or KI (BCFN_KI);
- Wheat flour (52%); water (41.8%); instant yeast (0.9%); and broccoli-impregnated with salt (5%)—non-iodised (BBN), KIO_3_ (BBN_KIO_3_) or KI (BBN_KI);
- Wheat flour (52%); water (41.8%); instant yeast (0.9%); and beetroot-impregnated with salt (5%)—non-iodised (BBTN), KIO_3_ (BBTN_KIO_3_) or KI (BBTN_KI);

After dough preparation, it rose for 1 h at room temperature. Ciabatta was formed and baked in a convection-steam oven at 220 °C for 20 min. The rolls were left to cool for about 1 h [38].

#### 2.1.3. Storage Conditions of Gnocchi Dumplings and Ciabatta Rolls

The cooked Gnocchi samples were stored at −21 °C for 100 days in vacuum-sealed, medium-density polyethene bags. The storage time at −21 °C was set at 100 days, taking into account the recommended safe storage time for cereal products in freezing conditions, which is 3 months (90 days) and added 10% above this range [39].

After baking the Ciabatta rolls, they were stored at −21 °C for 100 days and 21 °C for 8 days.

### 2.2. Methods

#### 2.2.1. Stability of Iodine

The following measurement points were selected for iodine:

Gnocchi dumplings—before heat treatment (“raw” dumplings), immediately after heat treatment (cooking) (“cooked” dumplings) and after storage of “cooked” dumplings at −21 °C for 100 days.

Ciabatta rolls—before heat treatment (“raw” dough), immediately after thermal processing (baked) and after storage at 21 °C for 8 days and at −21 °C for 100 days for baked samples.

The iodine content determinations for both types of products (Gnocchi dumplings and Ciabatta rolls) stored at −21 °C were performed on the 10, 20, 30, 40, 60, 80 and 100th day of storage. The iodine content in Ciabatta rolls during storage at 21 °C was determined on days 2, 3, 4, 5, 6, 7 and 8 of storage.

Total iodine content was determined using a macro-chemical method with potassium thiocyanate, as described by Kuhne [40] and Moxon and Dixon [41]. Waszkowiak and Szymandera-Buszka [42] previously described the details of the method.

#### 2.2.2. Stability of Thiamine

Thiamine content was determined in Ciabatta rolls.

The determinations were made in the “raw” dough before baking and set days of storage (−21 °C/100 days and 21 °C/8 days), similar to the determinations of iodine content. Thiamine was not determined in Gnocchi dumplings due to its low content in the raw materials, i.e., potatoes (0.042 mg/100 g [43]; 0.053 mg/100 g [own determinations]) and potato flour (0.0 mg/100 g) [43]. The samples were tested using the thiochromium method [33,44]. This analysis included quantitative changes in the free (thiamine hydrochloride) and bound (thiamine pyrophosphate) forms. A Jenway model 6200 fluorometer (Jenway, Stone, UK) (input filter with maximum 365 nm and output filter with maximum 435 nm) was used to measure the chromium fluorescence. All determinations were made in duplicate.

#### 2.2.3. Antioxidant Activity

The antioxidant activity of Gnocchi dumplings and Ciabatta rolls was analysed in “raw” dumplings and “raw” rolls after thermal processing (baking, cooking) and after storage.

The antioxidant activity of Gnocchi dumplings and Ciabatta rolls enriched with vegetables fortified with iodine samples was analysed immediately before and after baking and after storage. Extracts from samples were prepared by 2 h maceration with 80% ethanol (1:10 (*m*/*v*)) [45].

The antioxidant activity of all samples was based on the free radicals scavenging indices: the ABTS scavenging capability (ABTS^•+^) [46] and the DPPH scavenging capacity (DPPH^•^) [47,48].

#### 2.2.4. Statistical Methods

Analysis of the statistical dependencies between the means of antioxidant activity, thiamine contents and iodine contents (*p* < 0.01) was performed using Tukey’s multiple range test and PCA analysis. These analyses were calculated using the software STATISTICA PL 13.3 (StatSoft. Cracow, Poland). All indicators were analysed in 6 samples (3 measurements/2 independent series). Hypotheses were tested at α = 0.01. To prognosticate the dynamics of losses in iodine and thiamine in samples fortified with iodine throughout storage. The T_25%_ value was used. This calculation indicates the time in which the losses will amount to 25% [21].

## 3. Results

### 3.1. Changes in Nutritional Value During Thermal Processing of Gnocchi Dumplings and Ciabatta Rolls with Iodine Sources

#### 3.1.1. Iodine Stability

**Figure 1:**
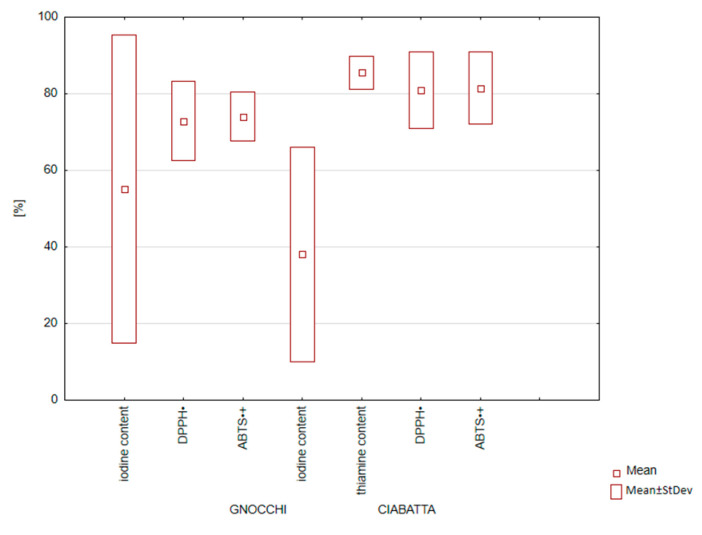
Box plot diagram of changes in nutritional value (thiamine, iodine, 2,2′-azinobis(3-ethylbenzothiazoline-6-sulfonic acid) (ABTS^•+^) radical cation and 2,2-diphenyl-1-picrylhydrazyl (DPPH^•^) radical) during thermal processing of Gnocchi dumplings and Ciabatta rolls with a vegetable fortified with iodine [content compared to samples before thermal processing—cooking/baking].

**Figure 2:**
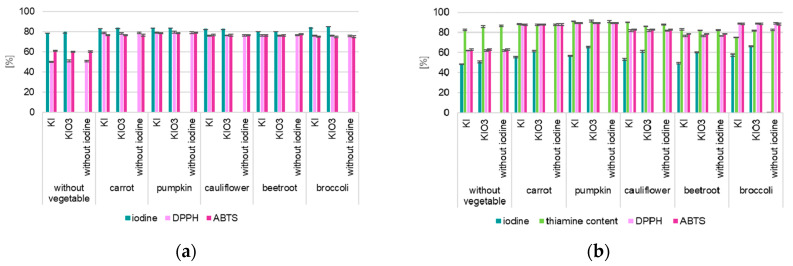
Changes in nutritional value (thiamine, iodine, 2,2′-azinobis(3-ethylbenzothiazoline-6-sulfonic acid) (ABTS^•+^) radical cation, 2,2-diphenyl-1-picrylhydrazyl (DPPH^•^) radical) during thermal processing of (**a**) Gnocchi dumplings and (**b**) Ciabatta rolls with vegetable fortified with iodine [content compared to samples before thermal processing—cooking/baking].

There was a statistically significant reduction in iodine content in products after heat treatment (Figure 1 and Figure 2). It concerned all designed products, both Gnocchi dumplings and Ciabatta rolls. Statistically significant (*p* < 0.001) greater iodine losses were confirmed when baking was used as heat treatment. Iodine losses amounted to 14–21% in Gnocchi dumplings and 33–51% in Ciabatta rolls.

Statistical analysis of predictors of variance models for changes in iodine content in Gnocchi dumplings and Ciabatta rolls with the addition of iodine-enriched dried vegetables (Table 1) confirmed the relationship between the form of iodine used for enrichment (KIO_3_/KI) and the stability of the iodine contained. This applied to bread and dumpling samples (F = 50.03 and F = 19,063.34; *p* < 0.001). These differences were higher by up to 8–11% for Ciabatta rolls. The highest losses were confirmed for this product with the addition of dried beetroot.

#### 3.1.2. Thiamine Stability

The Thiamine content was determined only in the samples of Ciabatta rolls.

This analysis confirmed thiamine losses at 8.85–21% (Figure 1 and Figure 2).

The most significant differences for a comparison between samples “without iodine” and “with iodine” were confirmed for samples without vegetables (5% for KI). Statistical analysis of predictors of variance models for changes in iodine content in Ciabatta rolls with the addition of iodine-enriched dried vegetables (Table 1) confirmed the relationship between the type of vegetable used for enrichment and the stability of the contained thiamine (F = 518.50, *p* < 0.001). Analysis of the obtained results (*p* < 0.001) confirmed the lowest thiamine losses in samples with the addition of dried pumpkin (9%) and the highest for broccoli and beetroot (18%).

#### 3.1.3. Antioxidant Activity

Analysis of the obtained research results confirmed a statistically significant effect of adding all analysed dried vegetables on the increased antioxidant activity of enriched products. Statistical analysis of predictors of variance models for changes in iodine content in Ciabatta rolls with the addition of iodine-enriched dried vegetables (Table 1) confirmed the relationship between the type of vegetable used for enrichment and the antioxidant activity of all samples regarding the capture of the ABTS cation radical (F = 7810.55, *p* < 0.001) and the DPPH radical (F = 2618.00, *p* < 0.001). Similar trends were confirmed for samples of Ciabatta dumplings. Statistical analysis (*p* < 0.001) confirmed the lack of correlation between the iodine content applied to the analysed vegetables and their antioxidant activity.

### 3.2. Changes in Nutritional Value After Storage of Gnocchi Dumplings and Ciabatta Rolls with Iodine Sources

#### 3.2.1. Iodine Stability

Analysis of changes in iodine content in Gnocchi dumplings and Ciabatta rolls with iodine-fortified vegetables confirmed decreased iodine content during storage.

**Figure 3:**
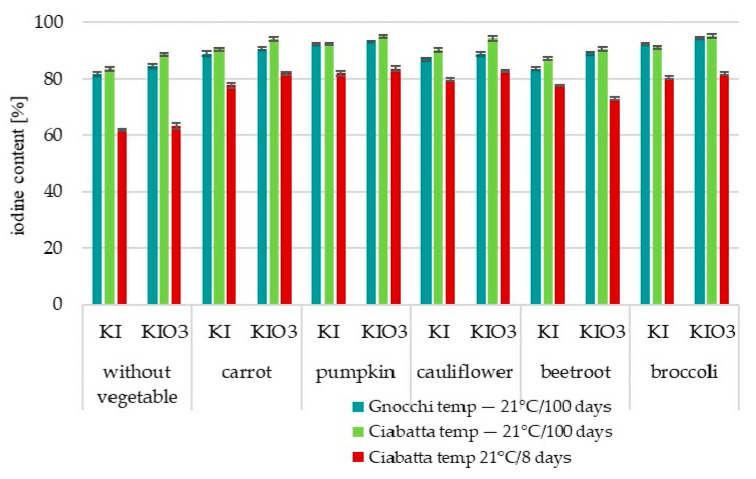
Changes in iodine content of Gnocchi dumplings and Ciabatta rolls with vegetables fortified with iodine after storage (content compared to samples after thermal processing—cooking/baking).

This was true for all variables. The analysis of changes in iodine content in Gnocchi dumplings stored at −21 °C/100 days (Figure 3, Table 2) confirmed iodine losses of 5 to 18% (Figure 3, Table 2). It was found that iodine from samples with iodine-fortified vegetables was more stable than from samples with only iodised salt (without vegetables) (Table S1). This concerned both analysed forms of iodine (KI, KIO_3_) (*p* < 0.001). The statistical analysis results confirmed the relationship between the type of fortified vegetable and the iodine content in stored samples (F = 0.65, *p* < 0.001). A statistically significantly higher iodine content was confirmed in samples by adding dried broccoli and pumpkin fortified with KI and KIO_3_ (5.5–7.7%). The lowest iodine content was confirmed in samples with dried beetroot KI fortified (16.4%). The samples can be ranked according to the increase in the rate of iodine transformation for KI and KIO_3_:

pumpkin < broccoli < carrot < cauliflower < beetroot < without vegetables.

Similar trends were confirmed for samples of Ciabatta rolls (Table 2). This concerned all analysed samples stored at 21 °C for 8 days (Figure 3) and 100 days at −21 °C. Iodine losses were determined at the level of 4 to 38%. Similarly to the Ciabatta roll samples, higher iodine stability was demonstrated in products enriched with iodine-fortified vegetables compared to samples without vegetables (with added iodised salt). This concerned both analysed forms of iodine (KI, KIO_3_) (*p* < 0.001). The statistical analysis results confirm a statistically significant effect of the type of vegetable used for enrichment on iodine stability both during storage at −21 °C (F = 43.71, *p* < 0.001) and 21 °C (F = 9.56, *p* < 0.001). The lowest iodine content was confirmed in samples by adding dried beetroot, regardless of the form of iodine. Analysis of the dynamics of changes in iodine content (T_25%_) (Table S1) confirmed the slowest rate of iodine losses for samples with added pumpkin and broccoli and the fastest for samples with added beetroot.

Similarly to Gnocchi samples, the samples can be ranked according to the increase in the rate of iodine transformation for KI and KIO_3_:

pumpkin < broccoli < carrot < cauliflower < beetroot < without vegetables.

**Table 2:** The characteristics of statistical analysis of variance predictors models for changes in iodine and thiamine content, and the ABTS^•+^ and the DPPH^•^ scavenging capacity of Ciabatta rolls enriched with iodine-fortified vegetables (KIO_3_/KI) storage (one-way ANOVA test).

Predictors	SS	df	MSE	F-Value	*p*-Value
IODINE					
GNOCCHI					
Storage −21 °C/100 days					
Vegetable type	591.37	5	118.27	0.65	0.00
Iodine form	31,027.57	2	15,513.79	908.15	0.00
CIABATTA					
Storage 21 °C/8 days					
Vegetable type	202.7	5	40.5	9.56	0.00
Iodine form	2.80	2	1.40	0.13	0.88
Storage −21 °C/100 days					
Vegetable type	386.60	5	77.3	43.71	0.00
Iodine form	3.30	2	1.60	0.29	0.76
THIAMINE					
CIABATTA					
Storage 21 °C/8 days					
Vegetable type	202.70	5	40.50	9.56	0.00
Iodine form	16.70	2	8.30	0.53	0.60
Storage −21 °C/100 days					
Vegetable type	386.60	5	77.30	43.71	0.00
Iodine form	10.20	2	5.10	0.19	0.83
DPPH^•^					
GNOCCHI					
Storage −21 °C/100 days					
Vegetable type	2452.79	5	490.56	49,431	0
Iodine form	0.00	2	0.00	0.00	1.00
CIABATTA					
Storage 21 °C/8 days					
Vegetable type	2623.20	5.00	524.60	2618.00	0.00
Iodine form	0.43	2.00	0.21	0.00	1.00
−21 °C/100 dni					
Vegetable type	4200.55	5	840.11	6820.10	0.00
Iodine form	0.67	2	0.33	0.00	1.00
ABTS^•+^					
GNOCCHI					
Storage −21 °C/100 days					
Vegetable type	2716.77	5	543.35	7232.70	0.00
Iodine form	0.15	2	0.08	0.00	1.00
CIABATTA					
Storage 21 °C/8 days					
Vegetable type	2188.86	5.00	437.77	7810.55	0.00
Iodine form	0.45	2	0.22	0.00	0.99
Storage −21 °C/100 days					
Vegetable type	4185.59	5	837.12	4489.4	0.00
Iodine form	171.85	2.00	85.93	0.38	0.69

SS—statistical significance; df—degrees of freedom; MSE—mean sum of squares.

#### 3.2.2. Thiamine Stability

**Figure 4:**
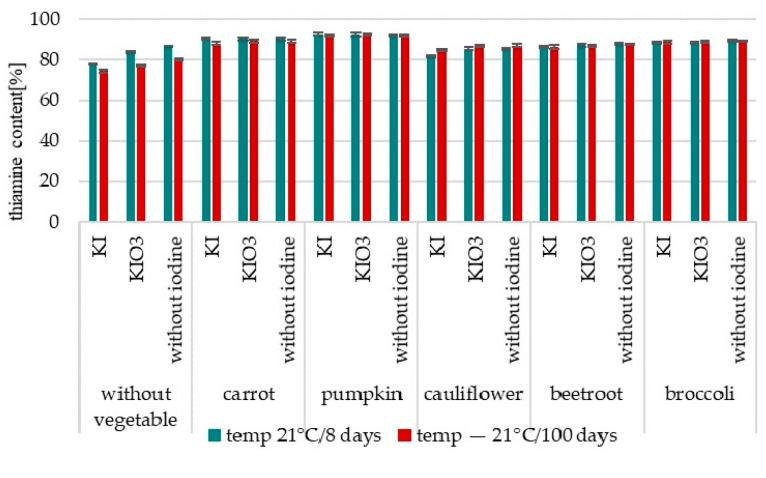
Changes in thiamine content of Gnocchi dumplings and Ciabatta rolls with vegetables fortified with thiamine after storage (content compared to samples after thermal processing—cooking/baking].

The analysis of changes in thiamine content in Ciabatta rolls with the addition of iodine-fortified and non-fortified vegetables and iodised salt confirmed a decrease in its content during storage. This concerned all analysed samples (Figure 4, Table S2). Thiamine losses were determined at the level of 7 to 25%. It was found (*p* < 0.001) higher stability of thiamine in products enriched with iodine-fortified vegetables compared to samples without vegetables added. In samples with the addition of iodised salt (without vegetables) was confirmed higher thiamine losses than with vegetables (up to 23%—for pumpkin).

Statistical analysis did not confirm the effect of the form (KI/KIO_3_) of the introduced iodine on the stability of the thiamine content, both during storage at 21 °C/8 days (*p* = 0.60) and −21 °C/100 days (*p* = 0.83) (Table 2).

Statistical analysis of predictors of variance models for changes in thiamine content confirmed a statistically significant effect of the type of fortified vegetable used to enrich Ciabatta rolls on the stability of the thiamine content, both during storage at −21 °C/100 days (F = 43.71, *p* < 0.001) and at 21 °C/8 days (F = 9.56, *p* < 0.001). Higher thiamine losses were confirmed in samples with the addition of iodised salt (without vegetables) than with vegetables (up to 23% for pumpkin). The smallest losses (7.38–8.04%) were confirmed for samples with the addition of both fortified and non-fortified pumpkins (*p* < 0.001). This concerned both storage variables (21 °C and −21 °C). The highest thiamine losses were confirmed for samples with the addition of dried cauliflower (13–18%). This concerned both analysed temperature variables (21 °C and −21 °C).

#### 3.2.3. Antioxidant Activity

**Figure 5:**
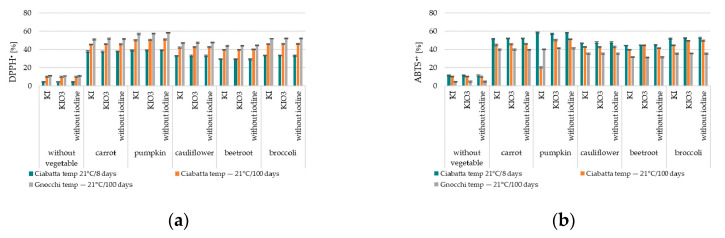
Changes in antioxidant activity value after storage of Gnocchi dumplings and Ciabatta rolls DPPH^•^ (**a**) and ABTS^•+^ (**b**) with a vegetable fortified with iodine (content compared to samples after thermal processing—cooking/baking].

The analysis of results confirmed a decrease in the antioxidant activity (ABTS^•+^, DPPH^•^) in all analysed samples at the level of 20–45% (Figure 5). This was true for both fortified and unfortified dried vegetables.

The highest antioxidant activity was confirmed for Gnocchi dumplings, regardless of the type of vegetable used, which should be explained by its higher amount in the product (24%) (Figure 5 and Table 2). The analysis of antioxidant activity (ABTS^•+^, DPPH^•^) stored at −21 °C/100 days of Gnocchi dumplings with the addition of iodine-fortified vegetables confirmed a decrease in this activity during the storage process at about 20–35% (Figure 5). Statistical analysis confirmed the relationship between the type of vegetable used for the enrichment of Gnocchi and the antioxidant activity of all samples concerning the scavenging of the cation radical ABTS (F = 7232.7, *p* < 0.001) and the DPPH radical (F = 49,431, *p* < 0.001). However, no relationship was confirmed between the form (KI/KIO_3_) and the antioxidant activity of all samples concerning the scavenging of the cation radical ABTS (*p* = 1.00) and the DPPH radical (*p* = 1.00). Similar trends were confirmed for the samples of Ciabatta rolls stored at 21 °C for 8 days and at −21 °C for 100 days.

The statistical analysis did not confirm the relationship between the form (KI/KIO_3_) and the antioxidant activity of all samples concerning the capture of the ABTS cation radical and the DPPH radical (Table 2).

### 3.3. Analysis of the Effectiveness of Using the Addition of Iodine-Fortified Dried Vegetables to Cereal Products (Gnocchi Dumplings and Ciabatta Rolls) Based on the RDA Coverage for Iodine

The designed Gnocchi dumplings were enriched with iodine by adding iodine-fortified (KI, KIO_3_) dried vegetables at level 24%.

**Figure 6:**
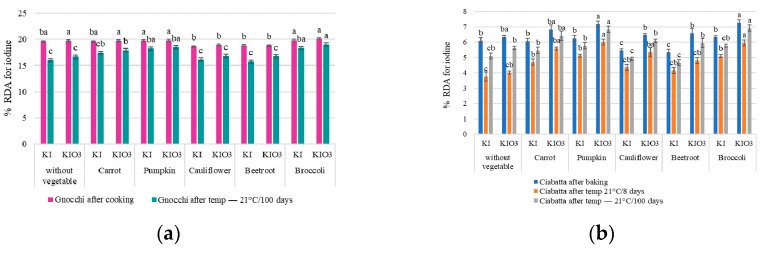
Degree of coverage [%] of the recommended daily intake of iodine (RDA) when consuming 100 g of iodine-enriched Gnocchi dumplings (**a**) and Ciabatta rolls (**b**) depending on the iodine carrier used, the form of iodine and the method of storage; different letters (a–c) within the same product group denote a significant difference at *p* < 0.05 (one-way ANOVA, and post hoc Tukey test).

Statistical analysis confirms that the degree of coverage [%] of the recommended daily intake of iodine (RDA—150 μg) with the consumption of 100 g of designed Gnocchi dumplings with the addition of iodine-fortified dried vegetables would cover the RDA for iodine at the level of 16.4–22.0%. Adding pumpkin, carrot, or broccoli as a matrix for the iodine introduced into the product allowed the RDA for iodine to be covered to the highest degree. Analysis of the type of vegetable used as a matrix for iodine enrichment of Gnocchi dumplings allowed confirmation of the estimated highest RDA coverage for iodine after consumption of dumplings enriched with dried carrot, pumpkin or broccoli. The designed Ciabatta rolls were enriched with iodine by adding 5% of iodine-fortified (KI, KIO_3_) dried vegetables. The analysis of the degree of coverage [%] of the recommended daily intake of iodine (RDA—150 μg) with the consumption of 100 g of iodine-enriched Ciabatta rolls (Figure 6) confirms vegetables would allow for the coverage of the RDA for iodine at a level of 3.5–7.0%.

## 4. Discussion

The experiments and statistical analysis found that the addition of iodine-fortified dried vegetables to flour products, such as Gnocchi dumplings and Ciabatta rolls, has a positive effect on the tested parameters of their nutritional value compared to the dried vegetables without the addition.

In the designed Ciabatta rolls and Gnocchi dumplings, an increased loss of iodine content was observed in the product after heat treatment compared to the product that was not subjected to heat treatment (“raw”). This phenomenon results from the sensitivity of iodine compounds to the effects of elevated temperature [31]. A decrease in iodine content in the product after heat treatment was also observed in this study [49]. All the samples analysed showed higher iodine stability in products enriched with iodine-fortified vegetables than samples with added iodised salt. The highest stability of iodine was confirmed in samples enriched by adding dried pumpkin or broccoli. This is confirmed by earlier studies on storing dried vegetables fortified with iodine [20].

This may be related to the product’s higher protein content and lower pH than other vegetables [50,51]. Previous data on impregnating vegetables with thiamine [21] confirm this.

The lower stability of iodine in samples with added beetroot may be related to this product’s higher sugar content [52,53,54]. Therefore, further research is required.

Pumpkin preparations contain a higher protein content [55,56,57,58], which may cause the formation of stable complexes of these components with iodine. Earlier studies on storing protein preparations fortified with iodine confirm this tendency [33,59]. The higher stability of iodine applied to broccoli may also indicate the formation of stable complexes with iodine [60].

The mechanism of iodine form transformation explains the high stability of KIO_3_. Iodate is reduced to iodide, and potassium iodide behaves like a simple ionic salt and is easily oxidised to I [61].

All the samples analysed also showed higher thiamine stability in products enriched with iodine-fortified vegetables than samples with added iodised salt. This may be related to the product’s higher protein content or phenolic compounds.

The earlier study confirms the increase in thiamine stability in systems containing protein preparations [33,34] or products rich in phenolic compounds [62]. There is a possibility of creating thiamine-active compound complexes, which increases thiamine stability.

Analysis of the research results also confirmed a statistically significant effect of adding all analysed dried vegetables on increasing the antioxidant activity of enriched products. Adding iodine did not reduce the antioxidant activity of all vegetables used. Previous data confirm the possibility of reducing antioxidant activity in systems containing iodine in the form of KIO_3_. However, this applies to much higher concentrations [37,63,64].

The designed cereal products (Ciabatta rolls and Gnocchi dumplings) with iodine-fortified dried vegetables are a good source of iodine in the diet.

Earlier studies [65,66,67,68] indicate that bread is a good target for fortification because it is consumed daily in many countries worldwide, and its production is simple and cheap. Similarly, pasta and noodles have become international products [69,70,71] and meet the above assumptions. Cereal products have already been successfully used in IDD (Iodine Deficiency Disorders) prevention programmes. In Denmark [72] and the Netherlands [73], iodised salt produced iodine-fortified bread. In both cases, prevention programmes involving fortified bread increased the UIC (Urinary Iodine Concentration) of the study population, and fortified bread was one of the main sources of iodine in the diet.

In future studies, it is worth developing the topic of ingredients contained in food (protein and phenolic compounds) and iodine stability.

Particular regard should be given to the mechanisms of complex formation that increase or decrease the nutritional value of iodine-enriched foods.

## 5. Conclusions

The designed cereal products (Ciabatta rolls and Gnocchi dumplings) with the addition of iodine-fortified dried vegetables are a good source of iodine in the diet and can be used as an element of IDD prevention.

Consuming 100 g of Ciabatta rolls (1 pc.) provides coverage of iodine demand at the level of ~6% RDA, while 100 g of Gnocchi dumplings (20 pcs.) ~19% RDA. The highest effectiveness was confirmed for dried pumpkins.

The type of iodine carrier (KI or KIO_3_) and the matrix (type of vegetable) for iodine introduced into cereal products such as Ciabatta rolls and Gnocchi dumplings affect the content of selected nutritional value indicators, such as iodine and thiamine content and antioxidant properties of the product.

## Figures and Tables

**Table 1:** The characteristic of statistical analysis of variance predictors models changes in iodine content, the ABTS^•+^ and the DPPH^•^ scavenging capacity of Gnocchi dumplings and Ciabatta rolls enriched with iodine-fortified vegetables (KIO_3_/KI) before and after the thermal processing (one-way ANOVA test).

Predictors	SS	df	MSE	F-Value	*p*-Value
GNOCCHI					
after the thermal processing					
IODINE					
Vegetable type	1083.10	5.00	216.62	1.06	0.40
Iodine form	9680.54	2.00	4840.27	19,063.34	0.00
DPPH^•^					
Vegetable type	2623.20	5.00	524.60	2618.00	0.00
Iodine form	188.40	2.00	94.20	1.73	0.19
ABTS^•+^					
Vegetable type	2188.86	5.00	437.77	7810.55	0.00
Iodine form	171.90	2.00	86.00	1.92	0.16
CIABATTA					
after the thermal processing					
IODINE					
Vegetable type	8.58	4.00	2.15	3.26	0.03
Iodine form	16.06	1.00	16.06	50.03	0.00
THIAMINE					
Vegetable type	0.01	5.00	0.00	20.08	0.00
Iodine form	0.00	2.00	0.00	0.51	0.60
DPPH^•^					
Vegetable type	2623.20	5.00	524.60	2618.00	0.00
Iodine form	188.40	2.00	94.20	1.73	0.19
ABTS^•+^					
Vegetable type	2188.86	5.00	437.77	7810.55	0.00
Iodine form	171.90	2.00	86.00	1.92	0.16

SS—statistical significance; df—degrees of freedom; MSE—mean sum of squares.

## Data Availability

The data presented in this study are available on request from the corresponding author.

## Supplementary Materials

The following supporting information can be downloaded at https://www.mdpi.com/article/10.3390/nu16244261/s1, Table S1: The characteristics of dynamics of changes in iodine content (mg kg^−1^) for the storage of Ciabatta rolls and Gnocchi dumplings enriched with iodine-fortified vegetables; Table S2: The characteristics of dynamics of changes in thiamine content (mg kg^−1^) for the storage of Ciabatta rolls enriched with iodine-fortified vegetables.
