# Supplementary material for: Nutritional Adequacy of Flour Product Enrichment with Iodine-Fortified Plant-Based Products
Source: Nutrients. 2024 Dec 10;16(24):4261. doi: 10.3390/nu16244261 (PMC11678579; doi:10.3390/nu16244261)
Supplement: Supplementary file 1 [file nutrients-16-04261-s001.zip › nutrients-3325152-supplementary.pdf]

Table S1. The characteristics of dynamics of changes in iodine content (mg kg−1) for the storage of Ciabatta rolls and Gnocchi dumplings enriched with iodine-fortified vegetables.

| | Dynamics of change in iodine content during 100 days of storage | | | | | | | | | |
|---|---|---|---|---|---|---|---|---|---|---|
| Vegetable variation | $T_{25\%}$ [days] | $R^2$ | *RMSE* | k | $A_0$* | $T_{25\%}$ [days] | $R^2$ | *RMSE* | k* | $A_0$* |
| | $KIO_3$ | | | | | KI | | | | |
| CIABATTA | | | | | | | | | | |
| Storage -21°C/100days | | | | | | | | | | |
| BWN | 213.67 | 0.964 | 0.061 | -0.0103 | $1.20*10^4$ | 152.59 | 0.984 | 0.056 | -0.0141 | $8.38*10^3$ |
| BCRN | 359.52 | 0.947 | 0.049 | -0.007 | $2.76*10^4$ | 238.91 | 0.978 | 0.043 | -0.009 | $8.78*10^3$ |
| BPN | 488.22 | 0.982 | 0.021 | -0.008 | $4.95*10^4$ | 307.35 | 0.947 | 0.740 | -0.007 | $1.26*10^4$ |
| BCFN | 416.90 | 0.985 | 0.054 | -0.005 | $1.51*10^4$ | 245.42 | 0.982 | 0.031 | -0.008 | $3.49*10^3$ |
| BBTN | 247.56 | 0.985 | 0.042 | -0.008 | $1.89*10^4$ | 183.22 | 0.985 | 0.047 | -0.005 | $3.06*10^3$ |
| BBN | 463.04 | 0.894 | 0.055 | -0.005 | $5.43*10^4$ | 263.47 | 0.985 | 0.032 | -0.008 | $1.34*10^4$ |
| Storage 21°C/8days | | | | | | | | | | |
| BWN | 6.26 | 0.837 | 0.509 | -0.4905 | $3.23*10^4$ | 5.76 | 0.867 | 0.479 | -0.5189 | $2.12*10^4$ |
| BCRN | 9.31 | 0.896 | 0.206 | -0.256 | $2.70*10^4$ | 7.85 | 0.904 | 0.208 | -0.272 | $8.48*10^3$ |
| BPN | 9.88 | 0.840 | 0.199 | -0.236 | $4.27*10^4$ | 9.37 | 0.876 | 0.199 | -0.225 | $1.09*10^4$ |
| BCFN | 9.26 | 0.835 | 0.242 | -0.221 | $1.37*10^4$ | 8.28 | 0.856 | 0.207 | -0.214 | $3.17*10^3$ |
| BBTN | 6.40 | 0.898 | 0.207 | -0.300 | $1.21*10^4$ | 7.94 | 0.912 | 0.168 | -0.230 | $2.78*10^3$ |
| BBN | 9.58 | 0.919 | 0.231 | -0.269 | $5.34*10^4$ | 8.87 | 0.908 | 0.189 | -0.253 | $1.30*10^5$ |
| GNOCCHI | | | | | | | | | | |
| Storage -21°C/100days | | | | | | | | | | |
| GWN | 172.45 | 0.975 | 0.203 | -0.041 | $6.53 *10^{12}$ | 142.33 | 0.987 | 0.176 | -0.045 | $6.53*10^{12}$ |
| GCRN | 279.39 | 0.988 | 0.091 | -0.026 | $7.47 *10^{12}$ | 220.97 | 0.990 | 0.101 | -0.033 | $6.80*10^{12}$ |
| GPN | 362.37 | 0.983 | 0.084 | -0.021 | $9.56 *10^{12}$ | 311.11 | 0.983 | 0.096 | -0.024 | $9.03*10^{12}$ |
| GCFN | 208.81 | 0.894 | 0.346 | -0.033 | $3.23 *10^{12}$ | 188.39 | 0.944 | 0.289 | -0.038 | $2.36*10^{12}$ |
| GBTN | 177.91 | 0.918 | 0.339 | -0.037 | $1.69 *10^{12}$ | 138.96 | 0.980 | 0.225 | -0.051 | $2.12*10^{12}$ |
| GBN | 410.09 | 0.935 | 0.141 | -0.017 | $1.34 *10^{13}$ | 293.37 | 0.961 | 0.152 | -0.024 | $8.59*10^{12}$ |

* $A_0$ - the initial content of iodine, k - decay constant [53].

Table S2. The characteristics of dynamics of changes in thiamine content (mg kg−1) for the storage of Ciabatta rolls enriched with iodine fortified vegetables.

| | **Dynamics of change in thiamine content during 100 days during storage** | | | | |
|---|---|---|---|---|---|
| Vegetable variation | **$T_{25\%}$ [days]** | $R^2$ | *RMSE* | **k** | **$A_0$*** |
| | | Storage -21°C/100days | | | |
| BWN_KI | 98.03 | 0.925 | 0.157 | -0.0005 | 1.23 |
| BWN_$KIO_3$ | 108.79 | 0.885 | 0.157 | -0.0005 | 1.23 |
| BWN | 115.46 | 0.918 | 0.155 | -0.0005 | 1.23 |
| BCRN_ KI | 205.71 | 0.978 | 0.154 | -0.0004 | 1.23 |
| BCRN_ $KIO_3$ | 231.81 | 0.968 | 0.150 | -0.0002 | 1.21 |
| BCRN | 222.56 | 0.935 | 0.148 | -0.0002 | 1.21 |
| BPN_KI | 310.09 | 0.890 | 0.002 | -0.0001 | 1.21 |
| BPN_ $KIO_3$ | 332.12 | 0.962 | 0.001 | -0.0002 | 1.24 |
| BPN | 326.80 | 0.954 | 0.001 | -0.0002 | 1.24 |
| BCFN_KI | 168.84 | 0.984 | 0.007 | -0.0003 | 1.22 |
| BCFN_$KIO_3$ | 200.68 | 0.965 | 0.001 | -0.0002 | 1.21 |
| BCFN | 207.22 | 0.972 | 0.001 | -0.0002 | 1.21 |
| BBTN_KI | 181.70 | 0.979 | 0.001 | -0.0002 | 1.19 |
| BBTN_$KIO_3$ | 200.29 | 0.945 | 0.002 | -0.0002 | 1.18 |
| BBTN | 203.38 | 0.951 | 0.002 | -0.0002 | 1.18 |
| BBN_KI | 208.74 | 0.922 | 0.002 | -0.0002 | 1.19 |
| BBN_KIO3 | 215.74 | 0.962 | 0.001 | -0.0002 | 1.22 |
| BBN | 219.46 | 0.922 | 0.002 | -0.0002 | 1.22 |
| | | Storage 21°C/8days | | | |
| BWN_KI | 8.63 | 0.922 | 0.005 | -0.0067 | 1.24 |
| BWN_KIO3 | 12.44 | 0.909 | 0.003 | -0.0042 | 1.23 |
| BWN | 15.84 | 0.908 | 0.003 | -0.0049 | 1.24 |
| BCRN_ KI | 20.90 | 0.911 | 0.017 | -0.0024 | 1.23 |
| BCRN_ KIO3 | 20.52 | 0.869 | 0.002 | -0.0022 | 1.21 |
| BCRN | 19.66 | 0.878 | 0.002 | -0.0023 | 1.21 |
| BPN_KI | 25.08 | 0.919 | 0.002 | -0.0020 | 1.22 |
| BPN_ KIO3 | 25.66 | 0.871 | 0.002 | -0.0019 | 1.24 |
| BPN | 26.11 | 0.911 | 0.001 | -0.0019 | 1.24 |
| BCFN_KI | 11.61 | 0.868 | 0.006 | -0.0052 | 1.23 |
| BCFN_KIO3 | 12.58 | 0.908 | 0.020 | -0.0041 | 1.22 |
| BCFN | 14.18 | 0.919 | 0.002 | -0.0033 | 1.22 |
| BBTN_KI | 14.57 | 0.912 | 0.005 | -0.0062 | 1.20 |
| BBTN_KIO3 | 15.91 | 0.834 | 0.003 | -0.0024 | 1.18 |
| BBTN | 16.28 | 0.891 | 0.004 | -0.0022 | 1.18 |
| BBN_KI | 16.00 | 0.841 | 0.004 | -0.0013 | 1.19 |
| BBN_KIO3 | 16.85 | 0.836 | 0.003 | -0.0028 | 1.22 |
| BBN | 17.18 | 0.812 | 0.009 | -0.0027 | 1.22 |

* $A_0$ - the initial content of thiamine, k - decay constant [19].
